# Differential stromal reprogramming in benign and malignant naturally occurring canine mammary tumours identifies disease-modulating stromal components

**DOI:** 10.1038/s41598-020-62354-8

**Published:** 2020-03-26

**Authors:** Parisa Amini, Sina Nassiri, Alexandra Malbon, Enni Markkanen

**Affiliations:** 1 Institute of Veterinary Pharmacology and Toxicology, Vetsuisse Faculty, University of Zürich, Zürich, Switzerland; 2 Bioinformatics Core Facility, SIB Swiss Institute of Bioinformatics, Lausanne, Switzerland; 3 Institute of Veterinary Pathology, Vetsuisse Faculty, University of Zürich, Zürich, Switzerland; 4 Present Address: The Royal (Dick) School of Veterinary Studies and The Roslin Institute Easter Bush Campus, Midlothian, EH25 9RG Scotland

**Keywords:** Cancer, Oncology

## Abstract

While cancer-associated stroma (CAS) in malignant tumours is well described, stromal changes in benign forms of naturally occurring tumours remain poorly characterized. Spontaneous canine mammary carcinomas (mCA) are viewed as excellent models of human mCA. We have recently reported highly conserved stromal reprogramming between canine and human mCA based on transcriptome analysis of laser-capture-microdissected FFPE specimen. To identify stromal changes between benign and malignant mammary tumours, we have analysed matched normal and adenoma-associated stroma (AAS) from 13 canine mammary adenomas and compared them to previous data from 15 canine mCA. Our analyses reveal distinct stromal reprogramming even in small benign tumours. While similarities between AAS and CAS exist, the stromal signature clearly distinguished adenomas from mCA. The distinction between AAS and CAS is further substantiated by differential enrichment in several hallmark signalling pathways as well as differential abundance in cellular composition. Finally, we identify COL11A1, VIT, CD74, HLA-DRA, STRA6, IGFBP4, PIGR, and TNIP1 as strongly discriminatory stromal genes between adenoma and mCA, and demonstrate their prognostic value for human breast cancer. Given the relevance of canine CAS as a model for the human disease, our approach identifies disease-modulating stromal components with implications for both human and canine breast cancer.

## Introduction

It is well accepted that the microenvironment surrounding cancer cells, the so-called cancer-associated stroma (CAS), plays a central role in both cancer initiation as well as its progression^1,2^. CAS is composed of various non-cancer cells, among them fibroblasts, immune cells, vascular cells, as well as extracellular matrix. CAS affects tumour cells in several ways: directly by promoting growth and survival of tumour cells, and also by stimulating their invasive and migratory capacity, thereby promoting invasion and metastasis^1,2^. Thus, CAS is considered to be a major determinant of tumour malignancy. However, in contrast to malignant tumours, it remains largely unexplored whether and what stromal changes occur in benign forms of naturally occurring tumours, and how stromal changes in benign neoplasms compare to that of malignant tumours in the same tissue. Such knowledge has the potential to help identify disease-promoting and/or suppressive features of the stroma and identify novel prognostic and therapeutic targets therein.

Close resemblance with regards to both pathophysiology and clinical aspects have positioned spontaneously occurring tumours in the domestic dog as valuable model to enhance understanding of tumour biology in both canine and human patients^3–5^. In particular, canine simple mammary carcinoma (mCA) are regarded as excellent models for human mCA, as they recapitulate the biology of human mCA both histologically and molecularly, and overcome several of the limitations of rodent tumour models^6–8^. Canine simple mCA are malignant epithelial neoplasms that infiltrate the surrounding tissue, thereby inducing a strong stromal response, and can give rise to metastases^9^. In contrast, canine simple mammary adenomas are well-demarcated, non-infiltrative benign mammary tumours generally associated with little fibrovascular supporting stroma^9^. Whether these benign adenomas can progress into more malignant forms, such as mCA, remains an unresolved controversy.

Given the central role of CAS in human cancer in general, and mCA in particular, it is likely to also play a key role in canine mammary tumours. To understand stromal reprogramming in canine mCA and how it compares to human mCA, we have previously analysed CAS reprogramming in formalin-fixed paraffin embedded (FFPE) breast cancer tissue using by laser-capture-microdissection (LCM) and quantitative PCR (RT-qPCR), and further advanced the approach to analyse LCM subsections by next-generation sequencing (RNAseq)^10,11^. Using this powerful RNAseq-driven approach, we have very recently assessed stromal reprogramming in a set of 15 canine mCA, and demonstrated strong molecular homology in stromal reprogramming between canine and human mCA, emphasizing the relevance of the canine model for the human disease also with regards to CAS reprogramming^12^.

For diagnostic and prognostic purposes, there is a high need for markers to reliably predict the clinical course of a tumour. This necessitates understanding what differentiates benign and malignant tumours on a molecular basis. Several studies have demonstrated differences between stromal expression patterns from human mCA *in situ* compared to invasive tumours, some of which can be used as predictive markers for disease^13,14^. However, data regarding stromal reprogramming in naturally occurring benign mammary tumours are inexistent. To analyse whether stromal reprogramming occurs in naturally occurring benign mammary tumours, and to compare stromal reprogramming between benign and malignant mammary tumours, we investigated 13 cases of canine mammary adenoma, and compared stromal reprogramming in canine adenoma to that in canine mCA^12^.

## Results

### Transcriptomic profiling of matched AAS and normal stroma from canine mammary adenomas isolated by laser-capture microdissection from FFPE specimens

**Figure 1:**
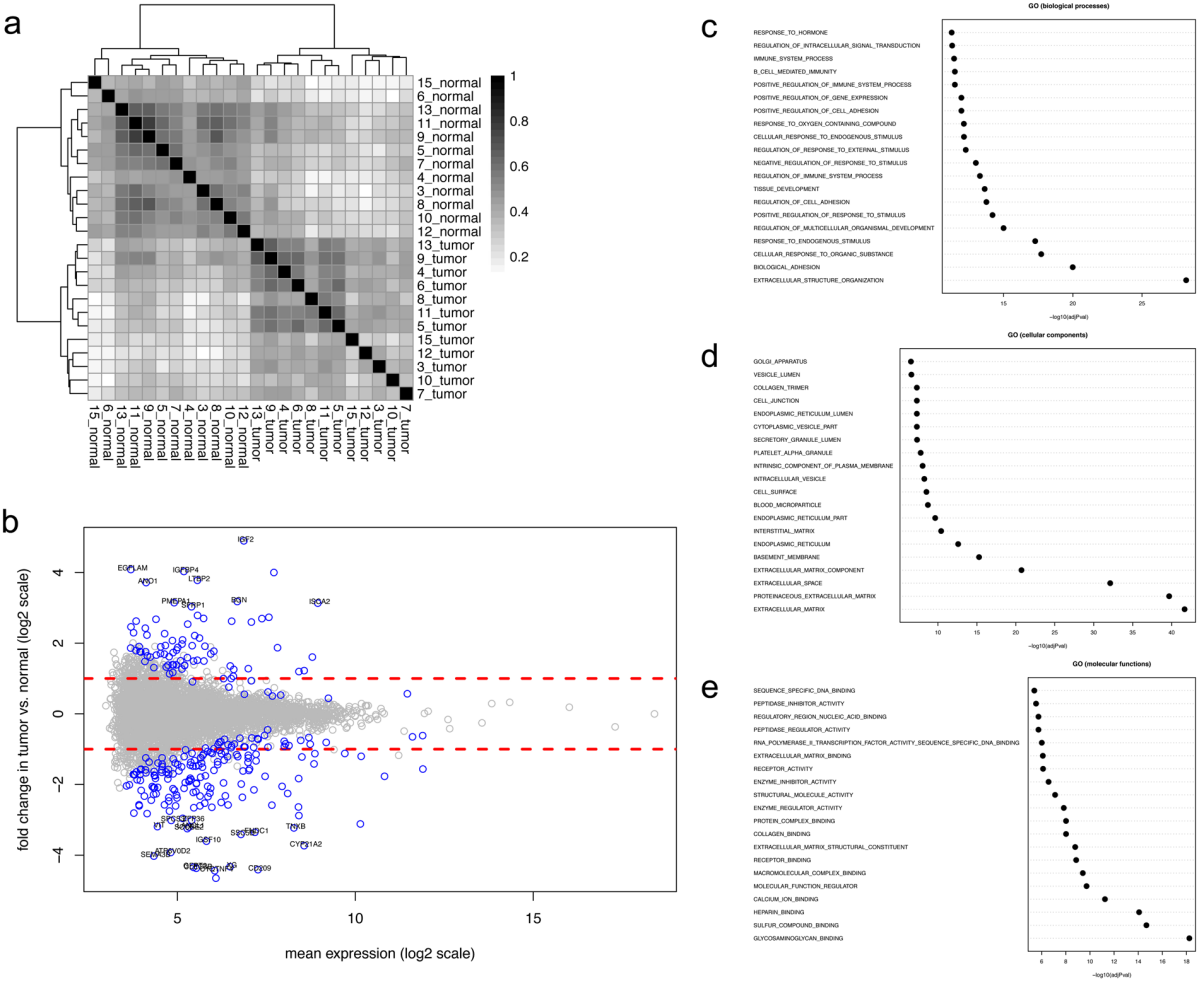
RNAseq-based transcriptomic analysis of cancer-associated stroma and matched normal stroma from 13 canine simple mammary adenoma. (**a**) Pairwise Pearson correlation analysis of adenoma-associated stroma (AAS) and normal stroma samples isolated from canine simple adenoma. The analysis was performed using all genes. Normal = normal stroma, Tumor = AAS. (**b**) Scatter plot of fold change versus mean expression highlighting differentially expressed genes in tumour stroma compared to normal stroma, using FC > 2 and FDR < 0.05 as cut-off values. (**c–e**) Top 20 over-represented Gene Ontology (GO) terms associated with biological processes (**c**), cellular components (**d**), and molecular functions (**e**) among genes significantly de-regulated in AAS compared to normal stroma.

To characterize stromal changes associated with canine simple adenomas, we isolated both adenoma-associated stroma (AAS) and matched normal stroma (i.e. stroma adjacent to unaltered mammary glands) from 13 FFPE samples of canine simple adenoma using our established protocol^11,12^. Of these, one pair had to be excluded due to extremely low sequencing depth (see methods for details). Patient characteristics for all adenoma cases that were included and representative images for tissue isolation can be found in Table 1 and Supplementary Figure 1. Pairwise sample-to-sample Pearson correlation analysis using all genes revealed a clear separation of normal stroma and AAS, demonstrating that AAS also undergoes a reprogramming that clearly differentiates it from normal stroma (Fig. 1a). Analysis of differentially expressed genes with a FDR cut-off of 0.05 and fold change threshold of 2 revealed 193 genes to be significantly deregulated in AAs compared to normal stroma, including 57 significantly up- and 136 significantly down-regulated genes (Fig. 1b and Supplementary Table 1). Over-representation analysis of GO terms associated with biological processes suggested changes in following GO categories: extracellular structure organisation, adhesion, response to organic substance and endogenous stimulus, regulation of multicellular organismal development, and responses related to the immune system (Fig. 1c). Moreover GO terms associated with cellular components revealed main changes pertaining to the extracellular matrix (Fig. 1d), which was also supported by GO terms associated with molecular functions, highlighting strong changes in binding of various ECM components (Fig. 1e).

**Table 1:** Overview of canine mammary simple adenoma cases included in this study.

Case #	Gender	Breed	Age (Years)	Simple Adenoma	Age of Sample
(Months)					
1	f	Rhodesian Ridgeback	8	yes	13
2	f	Deutscher Wachtelhund	6	yes	20
3	f	Golden Retriever	8	yes	24
4	f	Cocker Spaniel	8	yes	22
5	f	Coton de Tuléar	13	yes	29
6	f	Epagneul Breton	8	yes	6
7	f	Galgo Espanol	9	yes	7
8	f	Pinscher	11	yes	15
9	f	Basset	12	yes	9
10	f	Chihuahua	9	yes	8
11	f	Yorkshire Terrier	10	yes	11
12	f	Mittelpudel	11	yes	4
13	f	West Highland White Terrier	9	yes	4

Clinical data from dogs with simple mammary adenoma; Case # = case number as referred to within this study; age = age at excision of tumour; age of sample = time between initial tumour excision and sampling of stroma/RNA extraction.

**Figure 2:**
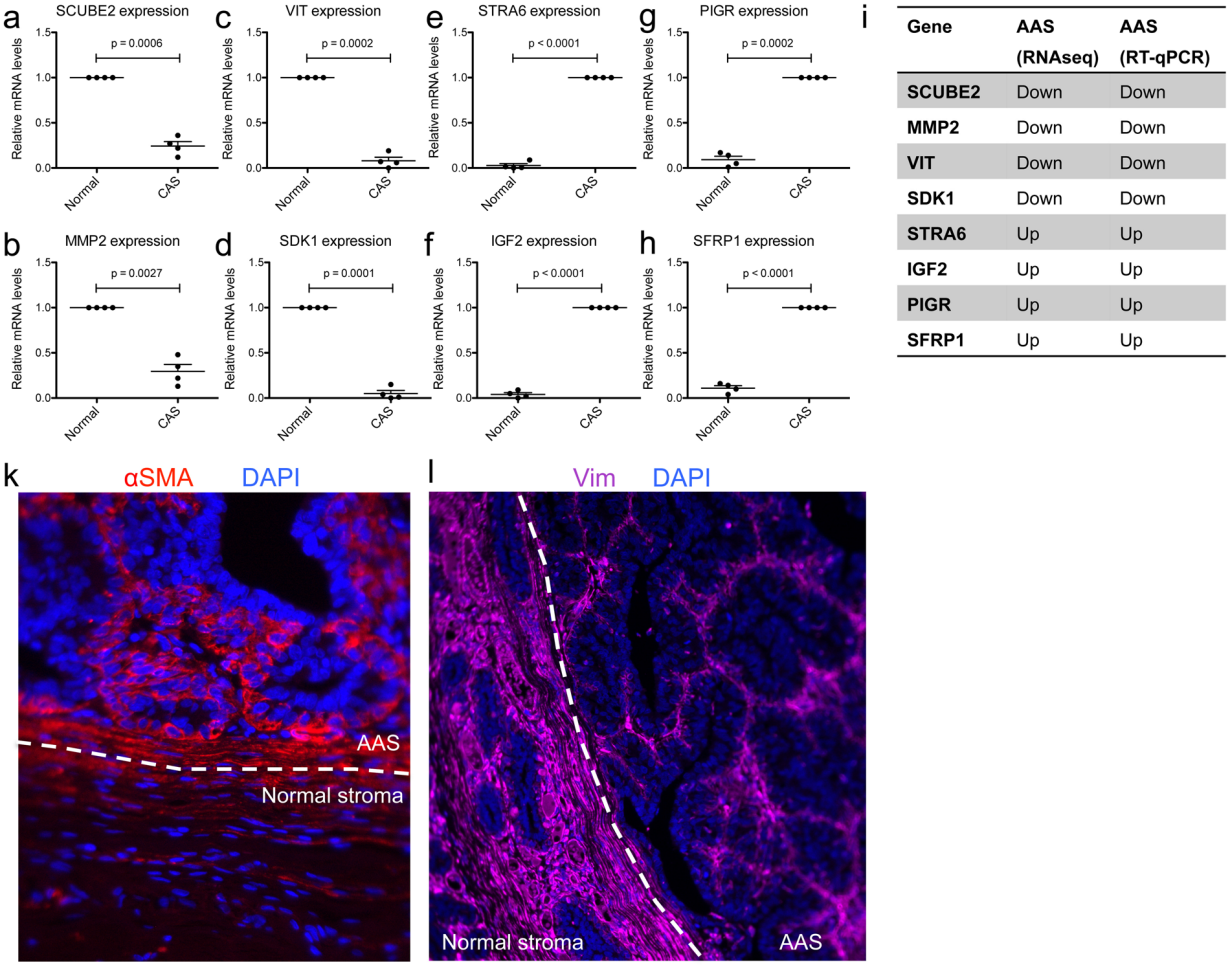
Validation of selected genes from the adenoma data by IF and RT-qPCR. (**a–h**) Relative mRNA levels of AAS-associated genes in normal stroma and AAS isolated by laser-capture microdissection, measured by RT-qPCR. **(a)**: SCUBE2; **(b)**: MMP2; **(c)**: VIT; **(d)**: SDK1; **(e)**: STRA6; **(f)**: IGF2; **(g)**: PIGR; **(h)**: SFRP1; Values are mean values ±SEM of four independent cases, normalized to expression levels in normal stroma (for SCUBE2, MMP2, VIT, and SDK1), or AAS (STRA6, IGF2, PIGR, and SFRP1), respectively. P-values were calculated using student’s t-test, and significance cutoff was set at p = 0.05. **(i)** Summary of the expression trends as detected by RT-qPCR and RNAseq. **(k**,**l)** Immunofluorescent staining of α-SMA (red) **(k)**, or Vimentin (purple) **(l)** in AAS and normal stroma of a representative canine simple mammary adenoma. Dapi staining (blue) visualizes cell nuclei. The dashed white line indicates the border between AAS and normal stroma.

Validation of RNAseq data was achieved through RT-qPCR of 8 strongly up- and down-regulated genes (SCUBE2, MMP2, VIT, SDK1, STRA6, IGF2, PIGR, and SFRP1), all of which showed significant expression changes consistent with RNAseq (Fig. 2a–i). Up-regulation of α-smooth muscle actin (α-SMA) in AAS compared to normal stroma was further evident on protein level by immunofluorescence (IF), in line with RNAseq results (ACTA2 log2 fold-change 0.875, p-value 0.0015; Fig. 2k). As myoepithelial cells tend to stain strongly for a-SMA in both normal and neoplastic mammary tissue, it cannot be excluded that there are myoepithelial cells as well as activated fibroblasts within the neoplasm in this image that stain positively for α-SMA. However, the solid areas of positive cells between acini in a simple adenoma are more suggestive of stromal cells. Furthermore, consistent with sequencing results (VIM log2 fold-change -1.044, p-value 2.58 E-08; Fig. 2l), vimentin expression decreased from normal stroma to AAS. Taken together, our data clearly demonstrate the occurrence of extensive stromal reprogramming in these benign naturally occurring tumours that is mainly driven by changes in the extracellular matrix, fibroblast activation and components of the immune system.

### The stromal signature distinguishes benign from malignant mammary tumours

**Figure 3:**
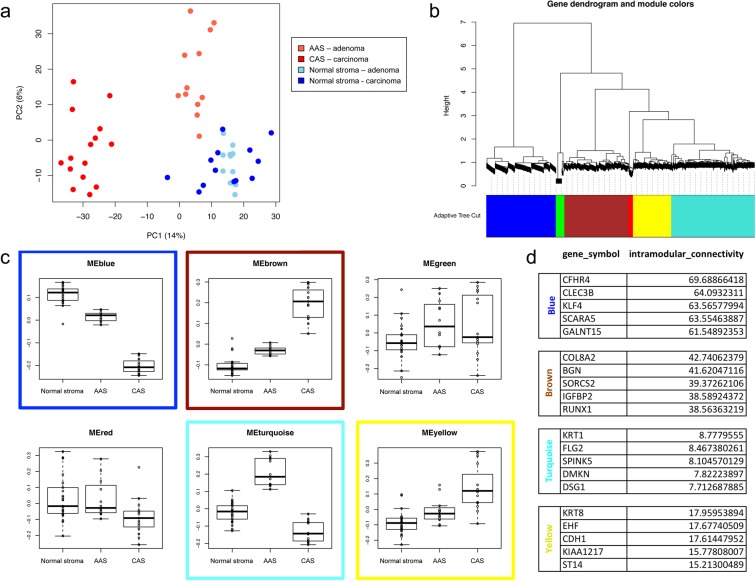
The stromal signature distinguishes benign from malignant canine mammary tumours. (**a**) Principal component analysis (PCA) of the batch-adjusted adenoma and mCA data combined. Defining each study as one batch, combined data was adjusted for potential batch effects under the assumption that normal stroma is similar between adenoma and mCA. Dark red = CAS from mCA, bright red = AAS from adenoma, dark blue = normal stroma from mCA cases, bright blue = normal stroma from adenoma cases. (**b**) Weighted gene coexpression network analysis reveals modules and hub genes associated with AAS and CAS. Modules of highly positively correlated genes withig the top 10% highly variable genes. (**c**) Association between module eigengenes and biological groups suggests four modules of interest: blue, brown, turquoise, and yellow. (**d**) List of top 5 hub genes from the selected modules and their intermodular connectivity values.

To understand how stromal reprogramming in benign canine mammary adenomas compares to malignant mCA, we juxtaposed our AAS dataset to a dataset of matched CAS and normal stroma from 15 canine mCA that we had obtained using the same methodology as previously reported^12^. As the histopathological appearance of normal, uninvolved stroma showed no difference between adenomas and mCA as expected, we merged the two data sets while adjusting for potential batch effects (see methods for details). Interestingly, PCA of the combined data showed three homogenous yet distinct clusters with the first two principal components clearly separating AAS and CAS from each other as well as from their normal counterparts, supporting the notion that stromal reprogramming is strongly influenced by the type of tumour (Fig. 3a). Furthermore, AAS seemed to be much more similar to normal stroma than CAS, suggesting that the stroma undergoes a gradual change during the development of malignant tumours.

Complementary to differential expression and gene set enrichment analysis which focus on individual genes and previously annotated gene sets, weighted gene co-expression network analysis (WGCNA) is a systems approach that allows for unbiased screening of genes based on their interconnectedness, thus revealing the inherent organization of the transcriptome that underlies the biology of interest and pointing out candidate targets and biomarkers for further investigation^15,16^. To start analysing transcriptional reprogramming of AAS and CAS, we applied WGCNA on a subset of highly variable genes, and identified six clusters of highly positively correlated genes, hereafter referred to as gene modules (Fig. 3b, Supplementary Figure 5, Supplementary Table 3). Closer inspection of module eigengenes as the summary of the expression pattern within each module revealed four potentially interesting modules whose expression significantly differed between normal stroma, AAS and CAS: modules blue, brown, turquoise and yellow (Fig. 3c). Module blue showed progressive down-regulation from normal to AAS to CAS, module turquoise showed opposite expression trends in AAS and CAS, whereas modules brown and yellow showed progressive overexpression from normal stroma to AAS to CAS. The observation that distinct modules were associated with AAS and CAS highlighted differential transcriptional reprograming between stroma in the proximity of adenomas and mCA. Finally, we examined intramodular connectivity to identify hub genes within each module of interest (Supplementary Table 3 for full list of genes). Figure 3d lists the top 5 genes with largest intramodular connectivity score serving as candidate hub genes for each module. The top 5 genes of the blue module were CFHR4, CLEC3B, KLF4, SCARA5 and GALNT15; for the brown module, these were COL8A2, SORCS2, BGN, RUNX1, and IGFBP2; for the turquoise module, we identified SPINK5, DSG1, FLG2, KRT1, and DMKN; and for module yellow the top 5 candidates consisted of CDH1, ST14, EHF, KRT8, and KIAA1217. These findings reveal the inherent structure of the transcriptome underlying AAS and CAS, and its modular distinction between adenoma and mCA. The identified hub genes serve as potential biomarkers or candidate targets for pharmaceutical intervention and will be subject of future studies.

**Figure 4:**
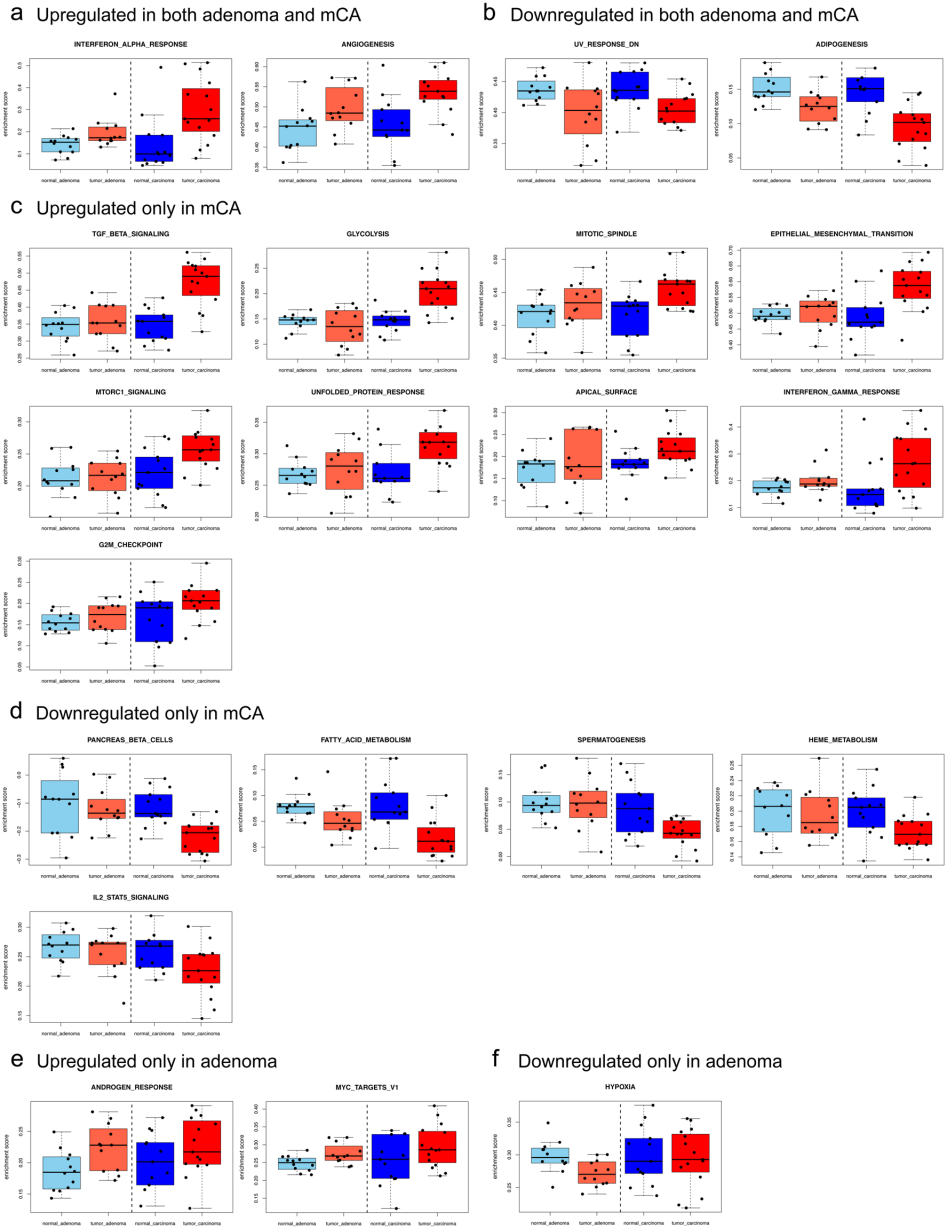
Single sample gene-set enrichment analysis of hallmark pathways among normal stroma, AAS and CAS. Pathways with an ANOVA p-value smaller than 0.05 are shown. (**a**) Pathways up-regulated in both AAS and CAS compared to normal stroma. (**b**) Pathways down-regulated in both AAS and CAS compared to normal stroma. (**c**) Pathways up-regulated only in CAS compared to normal stroma. (**d**) Pathways down-regulated only in CAS compared to normal stroma. (**e**) Pathways up-regulated only in AAS compared to normal stroma. (**f**) Pathways down-regulated only in AAS compared to normal stroma.

mCA differ in clinical and molecular aspects from adenomas. It is therefore reasonable to assume that distinct tumour-promoting pathways may be at play in the stroma from mCA compared to adenomas. Indeed, enrichment analysis of hallmark pathways among CAS from mCA, AAS from adenoma, and normal stroma revealed several hits that are significantly deregulated between normal stroma and stroma adjacent to adenoma and/or mCA. In both adenoma and mCA, interferon alpha response and angiogenesis were up-regulated, while UV response down and adipogenesis were down-regulated compared to normal stroma (Fig. 4a,b). In contrast, TGFbeta signalling, glycolysis, mitotic spindle, epithelial to mesenchymal transition, mTORC1 signalling, unfolded protein response, apical surface, interferon gamma response and G2M checkpoint showed significantly increased enrichment only in CAS (Fig. 4c), and pathways involving pancreas beta cells, fatty acid metabolism, spermatogenesis, heme metabolism and IL2-STAT5 signalling a significantly decreased enrichment only in CAS (Fig. 4d). Finally, significant up-regulation only in adenoma could be detected for androgen response and Myc targets V1, whereas decreased enrichment only in adenoma was detected for hypoxia. The full list of pathways and p-values can be found in Supplementary Table 4. Interestingly, for many of these pathways in AAS showed an intermediate enrichment between that of normal stroma and CAS, supporting the notion of progressive stromal adjustments to malignant transformation of the associated epithelium.

**Figure 5:**
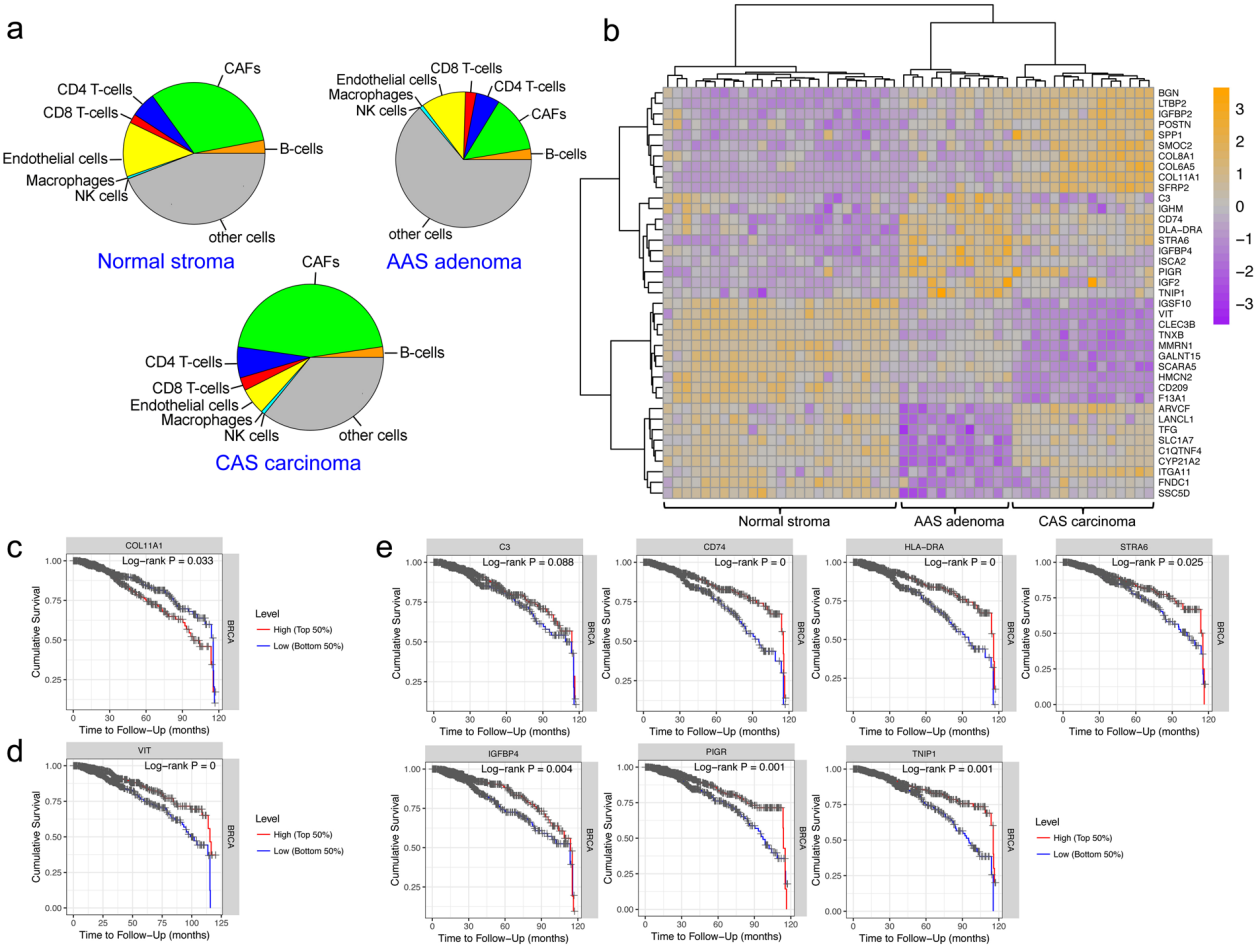
Most discriminatory features between AAS and CAS reveals genes with prognostic value for human breast cancer. (**a**) Pie charts summarising the average cellular composition of normal stroma, AAS and CAS as obtained from the EPIC algorithm. Pie area corresponds to average cellular fraction in the respective group. (**b**) Heatmap of 40 most discriminatory genes between AAS, CAS and normal stroma from both conditions, as revealed by PLSDA. Top 20 features with largest absolute loading were selected separately for the first and second components of the PLSDA model. Full list of PLSDA loadings can be found in Supplementary Table 2. (**c**–**e**) Kaplan-Meier plots of cumulative survival for the indicated genes to visualize survival differences between the upper and lower 50^th^ percentile of patients with a follow-up of 120 months.

Given that AAS and CAS are composed of various different types of non-cancer cells, the observed differences may also be influenced by the presence of differential cellular infiltrates in the stroma adjacent to adenoma or mCA. To further assess the contribution of changes in cellular composition to the observed transcriptional reprograming of AAS versus CAS, we utilized a previously established algorithm to estimate the proportion of main immune and stromal cells from CAS gene expression data^17^. Of the cell types quantified, cancer-associated fibroblasts (CAFs) and endothelial cells made up a large portion of the cellular composition of the stroma in all groups (Fig. 5a, Supplementary Figure 4). Of note, detection of CAFs in normal stroma most probably reflects the inherent difficulties in differentiating between fibroblasts and CAFs, and as such the CAFs detected by this methodology can be interpreted as ‘fibroblast-like cells’ either in normal stroma or cancer stroma. We found the fraction of CAFs to be higher in CAS than AAS. In contrast, the relative abundance of endothelial cells was lower in CAS compared to AAS. These findings suggest that reprograming of AAS and CAS as manifested in deregulation of genes and pathways is, at least in part, influenced by changes in the cellular composition of the stroma.

### Analysis of most discriminatory expression features between AAS and CAS reveals genes with prognostic value for human breast cancer

To identify characteristic features and assess the contribution of individual genes to the observed differences between normal stroma, AAS and CAS, we next used Partial Least Squares Discriminant Analysis (PLSDA). Intuitively, PLSDA can be viewed as a supervised extension of PCA, in which principal components are rotated such that maximum separation is achieved between groups of observations. The loading vector of the PLS components can then be used to extract most discriminatory features. The expression profile of 40 most discriminatory genes as revealed by PLS loadings (Fig. 5b, Supplementary Figure 2 for selected genes, and Supplementary Table 2 for the full list of genes) revealed several interesting clusters of genes: i) genes that are strongly up-regulated in CAS from mCA, but remain practically unchanged in AAS compared to normal stroma (IGFBP2, POSTN, COL11A1, SFRP2, and others); ii) genes whose expression is similar between CAS and normal stroma, but strongly increases in AAS (such as CD74, STRA6, and PIGR); iii) genes that are strongly down-regulated in CAS (e.g. IGSF10, HMCN2, and CLEC3B); and iv) and genes that are strongly down-regulated specifically in AAS (e.g. ARVCF, LANCL1, ITGA11, etc).

As CAS is known to exert a strong modulatory role on tumour progression, we reasoned that genes which are strongly differentially regulated between AAS and CAS could have a prognostic value for patient survival. Given the lack of long-term survival data for the canine cases included in this study, and the relevance of CAS from canine mCA for human breast cancer, we aimed to analyse the association of the human orthologues of the identified genes with survival. For this, we assessed the breast cancer (BRCA) subset of the TCGA database using the TIMER software^18^. Among the genes from cluster i) that are up-regulated in CAS, high (top 50%) COL11A1 expression was found to be significantly associated with worse survival (Fig. 5c). In contrast, for the genes in cluster iii), which are decreased in CAS, high VIT expression was found to be significantly associated with better survival (Fig. 5d). Strikingly, for genes in cluster ii), which are specifically up-regulated in AAS, high expression of 7 of the top 10 genes (C3, CD74, HLA-DRA, STRA6, IGFBP4, PIGR, and TNIP1) was strongly associated with better cumulative survival than tumours with low expression of these genes (Fig. 5e). Of note, IGHM expression could not be assessed in the dataset. Given their differential expression in benign versus malignant tumours as well as their association with a prognostic value, these genes are potentially interesting targets and their mechanistic relevance should be interrogated in future studies.

Taken together, our data demonstrate that the stromal signature clearly distinguishes benign adenomas from malignant canine mCA, suggesting that the stroma could be a discriminatory feature influencing the clinical course of the disease. Furthermore, our analyses specific perturbations of several candidate genes and signalling pathways as well as cell types to be associated with CAS of malignant tumours, and identify novel stromal targets associated with tumour malignancy and prognosis with relevance for human breast cancer.

## Discussion

CAS plays a key role in cancer initiation and progression in human cancer^1,2^. For diagnostic and therapeutic purposes, there is significant interest in understanding how stromal reactions between benign and malignant forms of the disease differ. This necessitates understanding what differentiates mCA from adenomas on a molecular basis. Stromal gene expression has indeed been shown to predict clinical outcome in invasive breast cancer^19,20^. Several studies have demonstrated differences between stromal expression patterns from human tumours *in situ* compared to those that display invasive properties, some of which can be used as predictive markers for disease (e.g.^13,14,21^). Similarly, studies using mouse models have analysed changes in stromal cell populations at different stages of mCA progression (e.g.^19^). To the best of our knowledge, however, there is no dataset (human or other) available that describes stromal reactions in naturally occurring benign mammary adenomas that could be used to compare to malignant mCA. Thus it remains unknown whether the stroma around benign adenomas undergoes reprogramming, and if so, what changes occur at the molecular level. As a consequence of this, it is unclear how these changes in benign neoplasms compare to malignant tumours of the same tissue. Such knowledge has the potential to help identify both disease-promoting and/or suppressing stromal features and identify novel prognostic and therapeutic targets therein. Given that canine mammary tumours are regarded as valuable model for human breast cancer, and the central role of CAS in human cancer in mCA, we have recently analysed stromal reprogramming in canine mCA, and demonstrated the presence of strong molecular homology in stromal reprogramming between canine and human mCA, emphasizing the relevance of the canine model for the human disease^10–12^. To understand whether stromal reprogramming also occurs in benign mammary tumours, and to compare stromal reprogramming between benign and malignant mammary tumours, we have now analysed stromal reprogramming in 13 cases of canine mammary adenoma and compared it to that in canine mCA.

Here we report that AAS from benign canine adenoma undergoes a reprogramming that clearly differentiates it from normal stroma, with major changes in GO terms related to extracellular structure organisation, adhesion, response to organic substance and endogenous stimulus, regulation of multicellular organismal development, immune responses, and the extracellular matrix (Fig. 1). These changes are consistent with fibroblast- and immune-cell driven remodelling of the tumour microenvironment, which are known to be heavily involved in tumour biology of mCA^1,2^. Indeed, we find clear signs of fibroblast activation and reprogramming, as evidenced by the increase in α-SMA and the decrease in vimentin by IF (Fig. 2), which is also seen in malignant canine mCA^10,12,22^. It is interesting that the stroma surrounding adenomas shows such a clear reprogramming, as these non-infiltrative benign mammary tumours are generally associated with little fibrovascular supporting stroma^9^. Of note, α-SMA reactive fibroblasts or slight up-regulation of several matrix-metalloproteinases and their inhibitors have been detected in benign and malignant lesions of the human breast^23–28^, as well as the canine mammary gland^22^. Hence, this first detailed glimpse into AAS surrounding spontaneous benign tumours of the breast suggests that stromal reprogramming is an early reaction to development of benign tumours and is characterized by strong transcriptional responses, the molecular minutiae of which have to be elucidated in future studies. The obvious stromal reprogramming adjacent to adenomas suggests a subset of alterations in the extracellular matrix to be early events in reactive stroma during initial tumour development, and not depend on tumour malignancy. Fibroblasts are the most abundant cells of the connective tissue, and responsible for production and maintenance the extracellular matrix^29^. Due to a very strong innate plasticity of fibroblasts, these cells are highly reactive towards changes in their environment, which gives rise to their inherent heterogeneity with regards to both phenotype and function. This makes them ideally adapted to fulfil very diverse roles ranging from maintenance of physical tissue support, wound healing to modulating inflammatory processes and supporting tumour growth. It thus seems that these changes in extracellular matrix composition might be mainly driven by fibroblast activation. Of note, this activation does not necessarily equal an increase in numbers of fibroblasts present, but may simply reflect their transcriptional status. It is well accepted, that the composition of the extracellular matrix strongly changes during tumour progression^2^. Recent advances have revealed a multitude of specific subpopulations of cancer-associated fibroblasts that display strong phenotypic diversity and functional heterogeneity (e.g.^29,30^). It will be of great interest now to investigate the detailed changes that discriminate the ‘early fibroblastic reaction’ that might be indiscriminate towards hyperplasia within a given epithelium from the reaction associated with malignant tumours to understand how the fibroblastic response changes in relation to tumour cell malignancy, and vice versa.

While not completely resolved, there is clear evidence that canine mammary gland tumours are a continuum from benign to malignant, supporting the comparison of canine mammary adenomas and mCA as different states of malignancy of the same disease^31^. Interestingly, PCA of the combined AAS and CAS shows adenoma-derived stroma to be much more similar to normal stroma than CAS from mCA, suggesting that the stroma undergoes a gradual change during the development of malignant tumors. This was further corroborated by WGCNA and enrichment analysis of hallmark pathways among CAS, AAS and normal stroma, where many of these pathways in AAS showed an intermediate enrichment between normal stroma and CAS (Figs. 3 and 4). With respect to the cellular composition, cancer-associated fibroblasts (CAFs) and endothelial cells made up a large portion of the cellular composition of all three groups (Fig. 5). We found the fraction of CAFs to be higher in CAS versus AAS, suggesting CAFs to be strong drivers towards tumour malignancy, consistent with current literature^29^. The relatively high number of CAFs in the normal stroma likely reflects the difficulties in differentiating between normal fibroblasts and CAFs based on subtle differences in their gene expression profiles. The lower relative abundance of endothelial cells in CAS compared to AAS is in line with the observation that malignant tumours often harbour large hypoxic or even necrotic areas due to insufficient vascular supply in relation to their strong proliferative properties. Indeed, a hypoxic tumour microenvironment and tumour progression are strongly linked^32^. Thus, we find that the stromal reprograming as manifested in deregulation of genes and pathways is, at least in part, also influenced by changes in the cellular composition of the stroma. Future work exploiting the composition of CAS and AAS, e.g. using single cell methods to simultaneously characterise changes in composition and state at the single cell resolution between benign and malignant tumours would help elucidating such changes further.

Identification of the hub genes (Fig. 4) SPINK5, DSG1, FLG2, KRT1, and DMKN the turquoise module, which is strongly decreased in CAS compared AAS, is highly interesting, since all of these genes have been strongly linked to maintenance of epithelial differentiation and integrity^33–37^. This suggests an important function of stromal reprogramming in destabilization of epithelial differentiation and integrity. For module yellow, which is progressively up-regulated from normal stroma to adenoma to mCA, the top candidate hub genes consisted of CDH1, ST14, EHF, KRT8, and KIAA1217, all of which have important roles in epithelial cells, and/or are associated with tumour malignancy^38–41^. Similarly, hub genes of module brown, COL8A2, SORCS2, BGN, RUNX1, and IGFBP2, displayed a progressive increase from normal stroma to AAS to CAS. These genes have important functions in the extracellular matrix and cell differentiation, and some have been associated with tumor progression or bad outcome in breast cancer^20,42–45^. Collectively, these findings reveal differences in transcriptional reprogramming of the stroma between benign and malignant breast tumours, and identify hub genes that could serve as potential biomarkers or candidate targets for pharmaceutical intervention. The detailed elucidation of the impact of these candidates to disease progression shall be subject of future studies.

Finally, by comparing stromal reprogramming in benign canine mammary adenomas to malignant mCA, we identified a list of gene targets that clearly distinguish AAS of benign adenomas from CAS in malignant mCA, further supporting the notion that the stroma has the potential of being a discriminatory feature influencing the clinical course of the disease (Fig. 5). Importantly, some of these differentially expressed genes show prognostic value for human breast cancer, demonstrating the value of comparative expression analyses across species. Interestingly, the gene cluster harbouring the most of these prognostic genes is overexpressed specifically in adenoma, arguing for a protective role for these genes against malignant progression. Given the involvement of many of these genes (such as C3, CD74, HLA-DR, STRA6, IGFBP4 and PIGR) in immune-mediated processes^46–50^, it is tempting to speculate that their up-regulation indicates immune-mediated tumour control that is lost in malignant tumours. Additionally, we found specific perturbations of e.g. EMT and glycolysis to be associated with CAS of malignant tumours (Fig. 4). EMT-related genes such as COL11A1, COL8A2, and ADAM12 that are overexpressed in mCA (Fig. 5) could thus be potential biomarkers for canine invasive mCA, similarly to human mCA^51–53^. In the glycolysis pathway, PLOD1/2, FUT8 and TSTA3 are deregulated genes that participate in metabolism and glycolytic processes, which can influence the malignant transformation of cells, tumour development and metastasis^54–56^. Further functional assessment of the differentially expressed targets and their association with tumour malignancy should be determined in future studies.

To conclude, we provide a first detailed view of stromal reprogramming in naturally occurring benign mammary adenomas, which demonstrates the occurrence of strong stromal reprogramming even in small benign tumours. Furthermore the stromal signature clearly distinguishes benign adenomas from malignant mCA, allowing identification of several hub genes as potential molecular drivers in the stroma. Given the relevance of canine CAS as a model for the human disease, our approach identifies potential stromal modulators of the disease with implications for human mCA.

## Methods

### Aim, case selection and tissue processing

We isolated AAS and matched normal stroma from FFPE tissue sections of canine mammary adenoma by LCM for transcriptome analysis by RNAseq. For this, 13 canine simple mammary adenoma samples were obtained from the Institute of Veterinary Pathology of the Vetsuisse Faculty Zürich (Table 1). All samples were archival formalin-fixed, paraffin-embedded tissue samples either from the Animal Hospital of Zurich or external referral cases from veterinarians practicing in Switzerland. Details regarding selection criteria are described in^10^. Paraffin blocks were routinely kept at room temperature. Tissue processing for LCM was performed as described in^11^. All cases were reviewed by a veterinary pathologist. Criteria for case selection included female dogs, simple mammary adenoma, and sufficient tumour stroma content for tissue isolation. Table 1 provides clinical details, such as age and breed of each patient, sample age and tumour type, for all cases included in the study.

### Laser-capture microdissection

Laser capture microdissection (LCM) for selective isolation of matched AAS and normal stroma was performed as previously described^10–12^. Areas for dissection were reviewed by a veterinary pathologist. Highly enriched populations of normal or tumour-associated stroma were identified and isolated according to the manufacturer’s protocol. Normal stroma samples were isolated from the same slides, from regions specified by a pathologist that were adjacent to unaltered mammary glands and presented no obvious alterations and were at least 2-4 mm away from the tumour, in accordance with established procedures^20^. Isolation of cells of interest was verified by microscopic examination of the LCM cap as well as the excised region after microdissection (Supplementary Figure 1).

### RNA isolation

RNA was isolated using the Covaris truXTRAC FFPE RNA kit and the Covaris E220 focused ultrasonicator as described in^11^. Details about RNA concentration, yield, and quality for adenoma samples can be found in Supplementary Table 5.

### Quantitative RT-PCR

Quantitative RT-PCR using Taqman primers was performed as described in^11^. Primers are detailed in Supplementary Table 6.

### Immunofluoresence

Immunofluorescence was performed as outlined in^12^. Antibodies and conditions used for immunofluorescence are detailed in Supplementary Table 7.

### RNA sequencing library preparation

10 ng of RNA from Elution 1 (E1) diluted to a concentration of 0.33 ng/μl in a total volume of 30 μl was submitted for next-generation RNA sequencing and analysed as outlined in^12^.

### Bioinformatics analyses

RNAseq quantification was performed with kallisto 0.44.0 with sequence-based bias correction using transcript sequences obtained from ENSEMBLE (CanFam3.1)^57^. Kallisto’s transcript-level estimates were further summarized at the gene-level using tximport 1.8.0 from Bioconductor^58^. Both raw data and gene-by-sample matrix of estimated counts have been deposited online and are publicly accessible from Gene Expression Omnibus (GEO) under accession number GSE135454. One of the normal stroma samples (14_normal) had extremely low sequencing depth and therefore was excluded along with its AAS pair (14_tumor) from downstream analyses. Carcinoma data was processed similarly as previously reported^12^, and can be accessed from GEO under GSE135183.

Prior to downstream analyses, lowly abundant genes were filtered out, and except for differential expression analysis, mean-variance trend was adjusted for using the variance-stabilizing transformation from DESeq. 2 1.22.0 package^59^. Pairwise sample Pearson correlation was computed in the adenoma data with top 10% most variable genes, and visualized using the pheatmap R Package^60^, with clustering distance and method set to Euclidean and ward.D2, respectively. Differentially expressed genes were identified using DESeq2 1.22.0^59^, with FDR = 0.05 and FoldChange=2 as significance thresholds. Over-representation analysis of Gene Ontology terms among significant genes was performed using the MSigDB webtool (http://software.broadinstitute.org/gsea).

For comparisons involving adenoma and carcinoma samples, the two datasets were merged using an intersection of genes present in both, followed by removal of lowly abundant genes. Treating each study as one batch and under the assumption that normal stroma is similar between adenoma and mCA, merged expression data was adjusted for potential batch effects using the ComBat empirical Bayes framework as implemented in the SVA 3.30.1 from Bioconductor^61^. To further mitigate technical noise, batch-corrected expression data was further adjusted for global differences across samples using quantile normalization as implemented in the limma 3.38.3 package from Bioconductor^62^. PLSDA was implemented using mixOmics 6.6.2 from Bioconductor^63^, with the number of components included in the model set to 2. Single-sample gene set enrichment analysis was performed using the ssGSEA functionality within GSVA 1.30.0 from Bioconductor^64^. In silico enumeration of cell types from bulk tissue gene expression data was performed using the EPIC algorithm^17^.

Coexpression network analysis was performed using WGCNA R package^65^. In brief, pairwise similarities were computed between top 10% most variable genes using biweight midcorrelation, and converted to an adjacency matrix with soft thresholding power set to 8. A signed topological dissimilarity matrix was then computed based on the adjacency matrix, and hierarchically clustered using the Ward’s minimum variance method. Following adaptive branch pruning of the clustering dendrogram, six gene modules were identified. To summarize the expression pattern within each module, module eigengene (defined as the 1^st^ principal component) was computed, and aligned along the average expression of the module to enhance interpretability. Finally, hub genes were identified based on the connectivity of nodes to other nodes within the same module, and visualized using Cytoscape^66^.

### Ethics approval and consent to participate

No animals were killed for the purpose of this research project, as the tissue analysed had been surgically removed in a curative setting with the verbal consent of the patient owners. According to the Swiss Animal Welfare Law Art. 3 c, Abs. 4 the preparation of tissues in the context of agricultural production, diagnostic or curative operations on the animal or for determining the health status of animal populations is not considered an animal experiment and, thus, does not require an animal experimentation license. All the used FFPE specimen were obtained for diagnostic reasons and do therefore not require a formal ethics approval, in full compliance with national guidelines.

## Supplementary information

Supplementary Information.

Supplementary Table 1

Supplementary Table 2

Supplementary Table 3

## Data Availability

Raw and processed sequencing data reported in this study have been deposited to Gene Expression Omnibus with the primary accession number GSE135454 and GSE135183. All other data supporting our findings is contained in the manuscript, in Supplementary Figures 1–5 and Supplementary Tables 1–7.
