# Supplementary material for: Differential stromal reprogramming in benign and malignant naturally occurring canine mammary tumours identifies disease-modulating stromal components
Source: Sci Rep. 2020 Mar 26;10:5506. doi: 10.1038/s41598-020-62354-8 (PMC7099087; doi:10.1038/s41598-020-62354-8)
Supplement: Supplementary file 1 — Supplementary Information. [file 41598_2020_62354_MOESM1_ESM.pdf]

Supplementary information

Differential stromal reprogramming in benign and malignant naturally occurring canine mammary tumours identifies disease-modulating stromal components

Parisa Amini, Sina Nassiri, Alexandra Malbon and Enni Markkanen

Supplementary Figure 1: Selective isolation of cancer-associated stroma and normal stroma from canine simple mammary adenomas by laser-capture-microdissection. Representative images of tissue mounted on the slide to validate proper isolation of stromal cells were taken at x 10 magnification before dissection (A, D), after dissection (B, E), and of the cap containing the excised tissue sections (C, F). Tissue is from one representative case: CAS (A-C) and normal stroma (D-F) from a simple mammary adenoma**.**

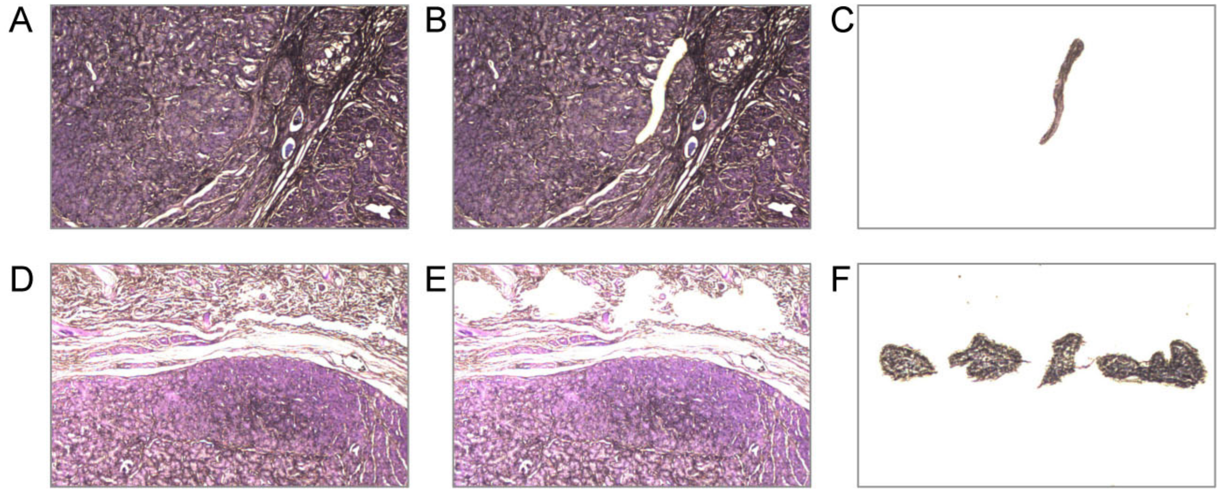

Supplementary Figure 2: Top 20 features with largest absolute loading were selected separately for the first (top) and second (bottom) components of the PLSDA model. Full list of PLSDA loadings can be found in Supplementary Table 3.

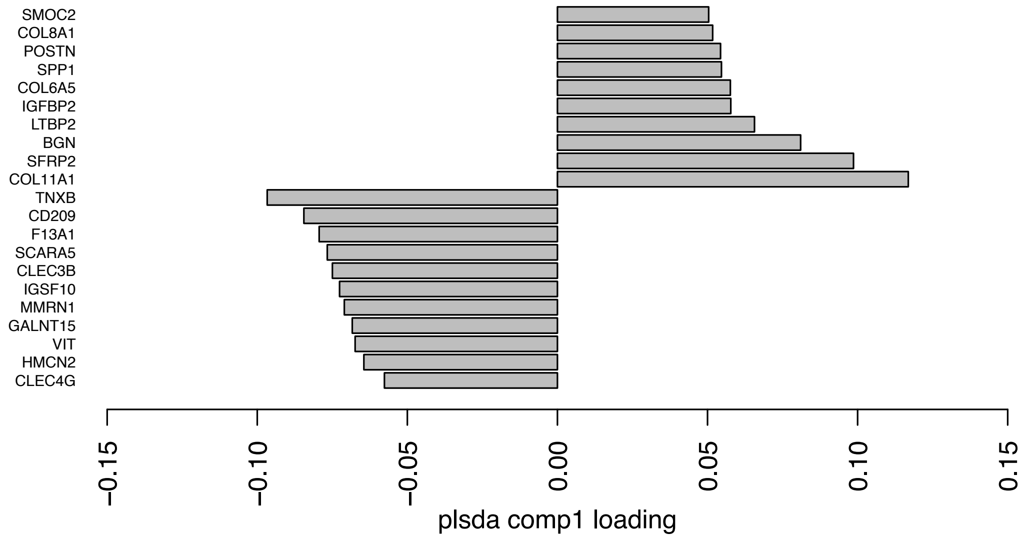

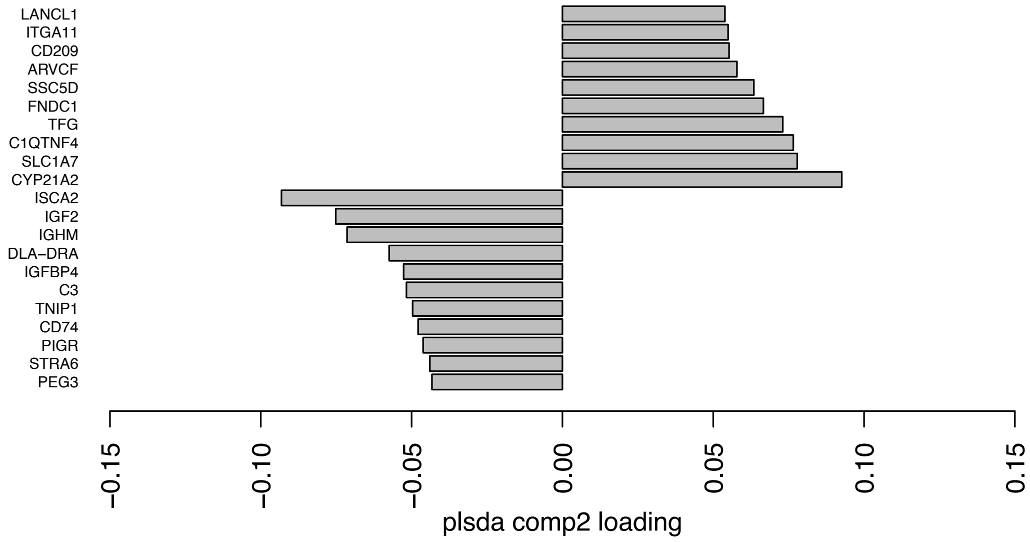

Supplementary Figure 3: GSEA analysis of hallmark pathways (MSigDB) in normal stroma, CAS from adenoma and CAS from carcinoma.

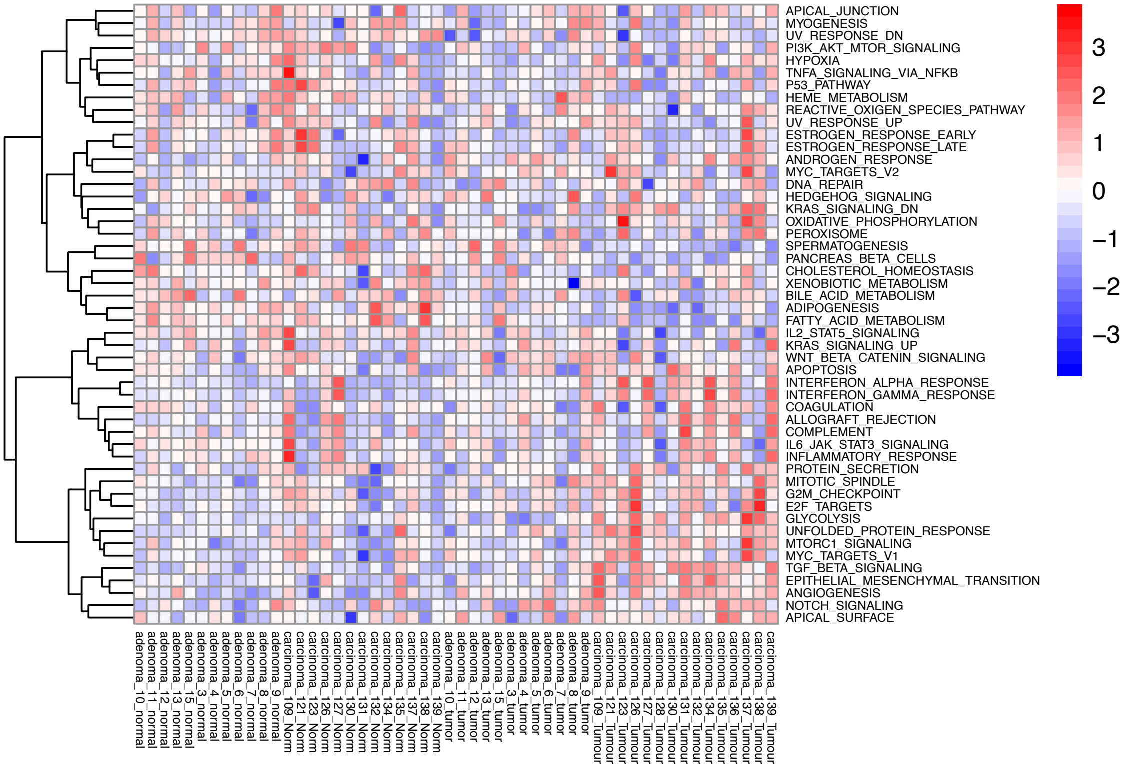

Supplementary Figure 4: EPIC in silico enumeration of cellular composition of CAS

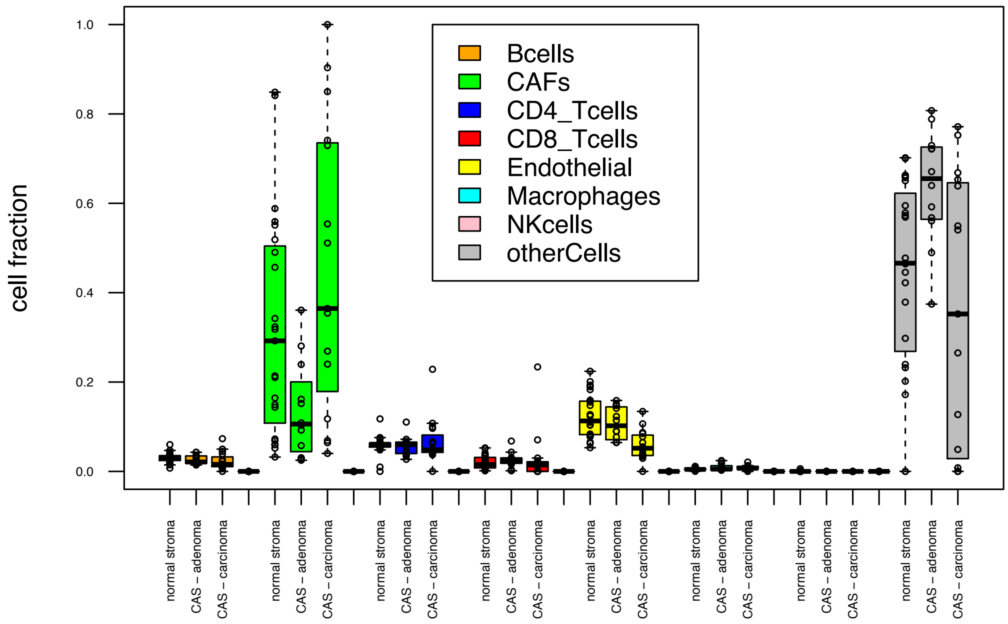

Supplementary Figure 5: Variable feature selection for network analysis

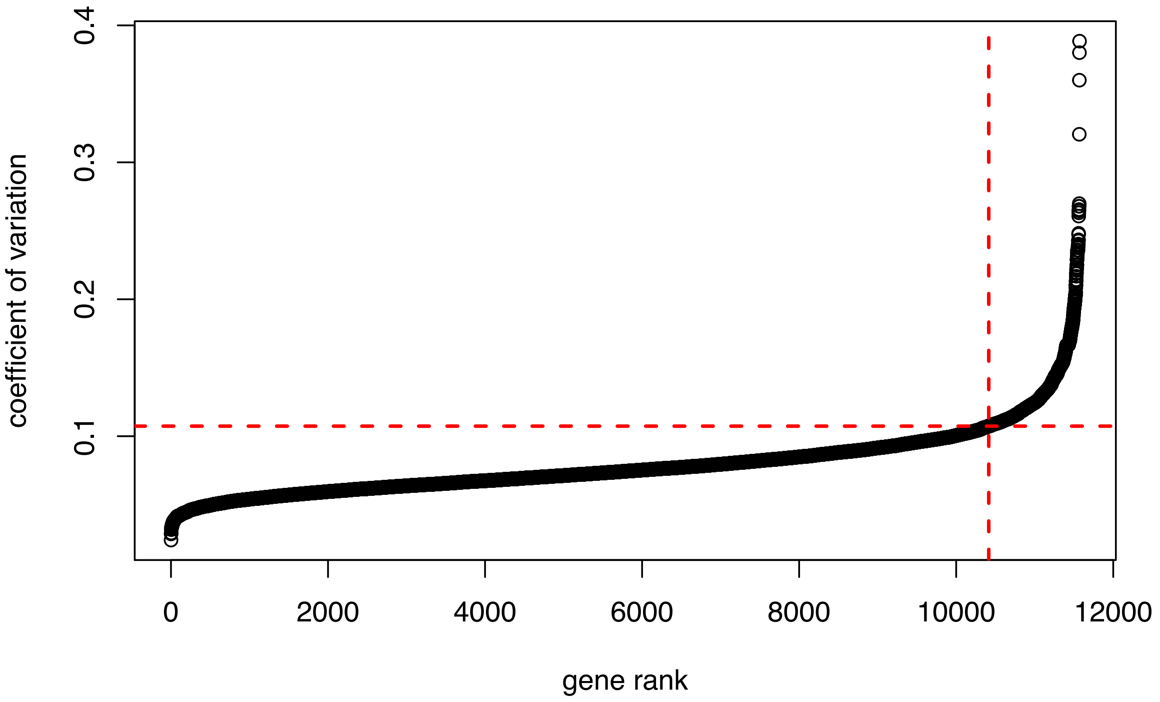

Supplementary Table 4. Full list of p-values of gene-set enrichment analysis of hallmark pathways among normal stroma and CAS from adenoma or carcinoma

	adenoma_pval	carcinoma_pval
TNFA_SIGNALING_VIA_NFKB	0.091225009	0.880288189
HYPOXIA	0.008929425	0.799796749
CHOLESTEROL_HOMEOSTASIS	0.056208273	0.410509971
MITOTIC_SPINDLE	0.203893797	0.001869307
WNT_BETA_CATENIN_SIGNALING	0.46958839	0.905847207
TGF_BETA_SIGNALING	0.536763773	6.91E-06
IL6_JAK_STAT3_SIGNALING	0.410822623	0.649569124
DNA_REPAIR	0.677265905	0.863982061
G2M_CHECKPOINT	0.310093653	0.043470099
APOPTOSIS	0.993133331	0.084035414
NOTCH_SIGNALING	0.246649532	0.055151004
ADIPOGENESIS	0.005339544	0.000676952
ESTROGEN_RESPONSE_EARLY	0.199795618	0.444005113
ESTROGEN_RESPONSE_LATE	0.316866387	0.569849348
ANDROGEN_RESPONSE	0.014052433	0.127837215
MYOGENESIS	0.67571902	0.517329184
PROTEIN_SECRETION	0.416144111	0.163817488
INTERFERON_ALPHA_RESPONSE	0.029115369	0.004877424
INTERFERON_GAMMA_RESPONSE	0.105217209	0.02781637
APICAL_JUNCTION	0.889770302	0.988692417
APICAL_SURFACE	0.530100985	0.023843854
HEDGEHOG_SIGNALING	0.351416795	0.844656239
COMPLEMENT	0.348714405	0.366042776
UNFOLDED_PROTEIN_RESPONSE	0.571178407	0.013942101
PI3K_AKT_MTOR_SIGNALING	0.487741598	0.560768866
MTORC1_SIGNALING	0.956783421	0.007261876
E2F_TARGETS	0.166149882	0.089826061
MYC_TARGETS_V1	0.015183233	0.111924655
MYC_TARGETS_V2	0.062183595	0.349752977
EPITHELIAL_MESENCHYMAL_TRANSITION	0.547431224	0.001950581
INFLAMMATORY_RESPONSE	0.204645778	0.95534455
XENOBIOTIC_METABOLISM	0.152611462	0.513423585
FATTY_ACID_METABOLISM	0.05403387	0.000594247
OXIDATIVE_PHOSPHORYLATION	0.55929747	0.315690328
GLYCOLYSIS	0.367900959	5.73E-05
REACTIVE_OXIGEN_SPECIES_PATHWAY	0.850309759	0.536672394
P53_PATHWAY	0.570645291	0.340571388
UV_RESPONSE_UP	0.860005806	0.983364436
UV_RESPONSE_DN	0.028818334	0.00715543
ANGIOGENESIS	0.027762817	0.001729331
HEME_METABOLISM	0.754103057	0.018290802
COAGULATION	0.216966724	0.355413854
IL2_STAT5_SIGNALING	0.367574559	0.027236925
BILE_ACID_METABOLISM	0.114570838	0.172076924
PEROXISOME	0.959449375	0.659502563
ALLOGRAFT_REJECTION	0.096668826	0.074699658
SPERMATOGENESIS	0.859904924	0.007598746

KRAS_SIGNALING_UP	0.498373265	0.689223979
KRAS_SIGNALING_DN	0.17421934	0.052067117
PANCREAS_BETA_CELLS	0.593631673	0.000147482

Supplementary Table 5. Summary of RNA yield and quality of samples used in this study.

Case #	Sample	Concentration (pg/ul)	Total yield (ng)	DV200 (%)
1	Normal	571	23670	52.58
	CAS	618	25100	64.72
2	Normal	380	17900	35.81
	CAS	934	38020	40.71
3	Normal	1050	37800	58.86
	CAS	1060	38010	69.14
4	Normal	573	26570	47.78
	CAS	2500	82080	72.20
5	Normal	748	25860	65.11
	CAS	347	15610	57.71
6	Normal	2750	96520	71.93
	CAS	1770	74300	71.51
7	Normal	1320	57240	63.67
	CAS	1010	30300	72.05
8	Normal	691	26630	65.73
	CAS	244	13640	68.90
9	Normal	493	15400	61.76
	CAS	546	24660	67.73
10	Normal	584	23560	63.68
	CAS	298	15300	62.90
11	Normal	608	23480	61.26
	CAS	425	18270	62.98
12	Normal	596	26680	76.23
	CAS	498	24260	70.42
13	Normal	705	36910	77.22
	CAS	600	27620	70.45

Supplementary Table 6. List of primers used for qRT-PCR. The "c" before each gene indicates that primers were designed to detect the canine isoforms of the intended targets.

Gene Target	Sequence	Amplicon Length (nt)	Taqman® Order Number or custom design reference
cGAPDH	Fw: 5'-GCTGCCAAATATGACGACATCA-3'<br>Rev: 5'-GTAGCCCAGGATGCCTTTGAG-3'<br>Probe: 5'-TCCCTCCGATGCCTGCTTCACTACCTT-3'	75	-
cPPIA	Manufacturer's proprietary information	92	Cf03986523_gH
cB2M	Manufacturer's proprietary information	87	Cf02659077_m1
cVIT	Manufacturer's proprietary information	68	Cf02657286_m1
cSTRA6	Manufacturer's proprietary information	61	Cf02661996_m1
cIGF2	Manufacturer's proprietary information	111	Cf02647135_m1
cPIGR	Manufacturer's proprietary information	70	Cf02625115_m1
cSFRP1	Manufacturer's proprietary information	69	Cf02654440_m1
cSCUBE2	Manufacturer's proprietary information	77	Cf02632274_m1
cMMP2	Manufacturer's proprietary information	58	Cf02623423_m1
cSDK1	Manufacturer's proprietary information	75	ARPRKXN

Supplementary Table 7. Antibodies and conditions used for immunofluorescence. Basic/acidic antigen retrieval indicate 20 min incubation in pH 9.0 EDTA buffer or pH 6.0 citrate buffer in a pressure cooker set to 98°, respectively.

**Primary antibody**					**Secondary antibody conditions**
Antigen	Type	Source	Antigen retrieval	Dilution And incubation	
SMA (anti human)	Monoclonal Mouse	M0851, Agilent	None	1:400, RT, 1h	goat anti mouse (Invitrogen A11005, 594) 1:400, 1 hour, RT
Vimentin (anti mouse)	Monoclonal Mouse	M7020, Agilent	Basic	1: 150, RT, 1h	goat anti mouse (Invitrogen A11005, 594) 1:400, 1 hour, RT
Collagen IV (anti mouse)	Monoclonal Mouse	Bio-Rad 2150-1470	Basic	1:50,4°C , over night	goat anti rabbit (Invitrogen A11012, 594) 1:400, 1 hour, RT
